# Genetic Association Between Sex Hormones, Serum Urate, and Gout: A Comprehensive Mendelian Randomization Study

**DOI:** 10.1155/ije/8839727

**Published:** 2026-04-20

**Authors:** Shiwei Li, Mengjuan Zhang, Xuemei Wang, Yadi Huang, Bo Huang, Meng Wang, Ming Liu, Jingqiu Cui

**Affiliations:** ^1^ Department of Endocrinology and Metabolism, Tianjin Medical University General Hospital, Tianjin, 300052, China, tjmugh.com.cn

**Keywords:** gout, Mendelian randomization, serum urate, sex difference, sex hormones, testosterone

## Abstract

**Background:**

Sex hormones have been associated with the risk of hyperuricemia (HUA) and gout in observational studies, but the potential causal relationships between sex hormones and risk of HUA and gout remain largely unclear. This study aimed to further investigate the mechanism underlying the causality between sex hormones, HUA, and gout in different genders.

**Methods:**

We used sex‐specific genome‐wide association study (GWAS) data for European populations. The genetic correlation between sex hormones and serum urate or gout was analyzed using linkage disequilibrium score regression (LDSC). Two‐sample Mendelian randomization (MR) analysis assessed the causality between sex hormones (total testosterone [ToT], bioavailable testosterone [BioT], SHBG, estradiol, age at menarche, and age at menopause) and serum urate or gout, followed by sex‐stratified analysis. Colocalization analysis examined shared genetic variants between sex hormones and outcomes. Sensitivity analyses were additionally performed to address potential pleiotropy. A two‐step MR was conducted to evaluate the mediation effects of circulating metabolites linking sex hormones with serum urate.

**Results:**

Genetic correlation analysis revealed moderate associations between sex hormones and serum urate/gout. MR analysis indicated a significant association between genetically predicted ToT and increased serum urate levels among women (*β*: 0.035, 95% CI: 0.006–0.063, and *p* = 0.018). Mediation analysis suggested that androstenediol (3β, 17β) disulfate (2) partially mediated the effect of ToT on serum urate levels, with a mediation proportion of 28.4%.

**Conclusions:**

These findings provide robust evidence that sex hormones influence urate metabolism with significant sex‐specific differences, suggesting tailored interventions for HUA, especially in European women with hyperandrogenism.

## 1. Introduction

Urate is the terminal metabolic product of purine metabolism in humans. Elevated levels of serum uric acid (> 420 μmol/L) are defined as hyperuricemia (HUA) [1]. HUA may cause the weakly soluble urate to become saturated and precipitate as monosodium urate (MSU) crystals. MSU crystals can deposit in the synovial fluid of joints, leading to gout, which is the most common form of inflammatory arthritis [2]. The prevalence of HUA in the general population ranges from 17.4% to 20.1% in different countries and regions [3, 4]. Unfortunately, the incidence has significantly increased in recent years, making it another common metabolic disease after diabetes. Additionally, a significant sex difference has been observed in the prevalence of HUA and gout, with the incidence in men being significantly higher than in women [5]. Population‐based studies in Europe and North America have reported a male‐to‐female prevalence ratio ranging from 2:1 to 4:1 [4, 6]. The observed sexual dimorphism may be attributed to the effects of sex‐specific hormones. Estrogen, for example, is known to lower serum urate levels by enhancing intestinal urate excretion, offering a protective effect against HUA [7]. The significant sex difference in the incidence of HUA and gout, along with their extreme rarity in premenopausal women and hypogonadal men, has led to the hypothesis that the prevalence of HUA and gout may be related to sex hormones [8, 9].

Sex hormones are a class of steroid hormones primarily synthesized and secreted by the gonads. Additionally, the adrenal glands can also secrete sex hormones, mainly androgens. Sex hormone‐binding globulin (SHBG) is a circulating glycoprotein secreted by the liver and is considered an important transport protein and regulator of sex hormones, mainly binding to androgens and estradiol to enhance or inhibit their effects [10]. Sex hormones and SHBG have been associated with a wide range of health outcomes, such as type 2 diabetes [11], non‐alcoholic fatty liver disease [12] and Alzheimer’s disease [13]. The connection between sex hormones and urate metabolism has also been supported by various epidemiological and genetic studies. For instance, androgen excess in conditions such as polycystic ovary syndrome has been associated with higher serum urate levels and an increased prevalence of HUA [14]. Although many studies have reported sex differences in the incidence of HUA and gout [6, 15], research progress on the relationship between sex hormones and HUA or gout has been slow in recent years, leading to ongoing controversy regarding the findings and underlying mechanisms [16]. In addition, most of these studies have been limited by small sample sizes, cross‐sectional designs, or short follow‐up periods [17, 18].

Mendelian randomization (MR) is a methodological approach in epidemiology that employs genetic variants as instrumental variables (IVs) to enhance causal inference [19]. Recently, there has been a growing interest in analytical frameworks that integrate genetic correlation with MR analysis [20, 21], as well as MR in conjunction with colocalization analysis [22, 23], to elucidate causal relationships more effectively.

Therefore, we have combined genetic correlation, MR, and colocalization methods to explore the causal relationship between sex hormones and serum urate levels or gout. This study is the first to adopt such an integrative framework, synthesizing genetic evidence from these three methodologies to offer a comprehensive understanding of how sex hormones influence serum urate and gout in European populations. To further investigate the mechanism underlying the causality among them, our study is followed by a sophisticated two‐step MR analysis employing an array of 1400 blood metabolites to unravel the potential pathways through which sex hormones exert their influence on serum urate/gout, thereby augmenting our understanding of the sex differences among HUA and gout.

## 2. Materials and Methods

### 2.1. Study Design

**FIGURE 1:**
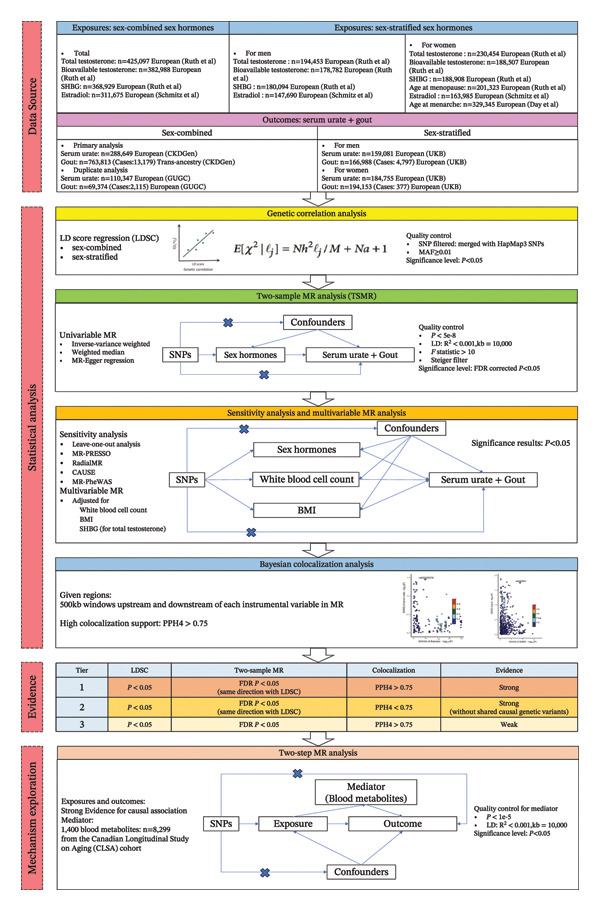
Overview of the study design and process. Abbreviations: SHBG, sex hormone‐binding globulin; UKB, UK Biobank; LD, linkage disequilibrium; SNP, single‐nucleotide polymorphism; MAF, minor allele frequency; MR, Mendelian randomization; FDR, false discovery rate; BMI, body mass index.

Our study was based on sex‐specific summary‐level genome‐wide association study (GWAS) data publicly available for European populations to investigate the potential causal associations between sex hormones (including age at menarche/menopause among women) and the level of serum urate or risk of gout. Genome‐wide linkage disequilibrium score regression (LDSC) was used to assess the genetic correlation between sex hormones and outcomes [24]. A two‐sample MR analysis was performed to explore the causal relationship between sex hormones and outcomes. In addition, a sex‐stratified MR analysis was conducted to explore sex‐specific causality among men and women. Colocalization analysis was employed to examine the shared local genetic architecture between sex hormones and the corresponding outcomes, as well as to determine whether the observed causal associations occurred by chance [25]. An overview of our analytical design and processes is illustrated in Figure 1. This study followed the STROBE‐MR guidelines [26], and the STROBE‐MR checklist is shown in Additional File 1: Table S1 [27].

All research protocols involving human subjects received approval from the local institutional review board, and informed consent was obtained from all participants. Additionally, all data utilized in this study are publicly accessible.

### 2.2. Data Sources

#### 2.2.1. GWAS Data for Sex Hormones

The GWAS summary‐level data of single‐nucleotide polymorphisms (SNPs) associated with exposures (total testosterone [ToT], bio‐available testosterone [BioT], SHBG, estradiol [binary only], age at menarche, and age at menopause) were released in the published study [28–31] (Additional File 1: Table S2 [27]). Due to lack of estradiol GWAS of both sex, we performed a fixed‐effect meta‐analysis using Meta‐Analysis Tool for genome‐wide association scans (METAL, Version 2011‐03‐25) to establish the effect estimates of estradiol for the combined male and female population (from here on referred to as the total population) [32]. When multiple summary statistics from GWAS were available for a particular exposure, the study with the largest sample size was selected. All GWAS summary statistics for exposures were at a minimum adjusted for age and sex, except for the variables of age at menarche/menopause. Furthermore, the GWAS data for SHBG and estradiol were adjusted for body mass index (BMI), as BMI is associated with both SHBG and estradiol, potentially confounding the relationship with serum urate and gout [33–35].

Given that testosterone exhibits a strong genetic correlation with SHBG, it becomes challenging to differentiate the effects of testosterone from those of SHBG. BioT is considered a more sensitive marker of active testosterone in men [36], showing significantly less correlation with SHBG compared with TT (TT *r* ^2^ 0.73 and BioT *r* ^2^ 0.05) [28]. In women, TT has a lower correlation with SHBG than BioT (TT *r* ^2^ 0.06 and BioT *r* ^2^ 0.74) [28]. Overall, a total of 182 independent SNPs were identified for predicting TT in men, while 216 SNPs were selected for women. Similarly, 93 independent SNPs were extracted to estimate BioT for both men and women.

#### 2.2.2. GWAS Data for Serum Urate and Gout

Comprehensive details of each outcome from the GWAS are presented in Additional File 1: Table S2 [27]. The genetic association data pertaining to gout and serum urate were sourced from the meta‐analysis conducted by Tin et al. [37]. The original publication outlines the demographic information, genotyping methods, and urate assay techniques utilized in each study. The dataset included 13,179 gout cases (identified through self‐reports, urate‐lowering medications, or ICD codes) and 750,634 control subjects. Notably, half of the gout cases originated from the UK Biobank, with 98% of the cases representing individuals of European ancestry. In this study, urate data (measured in mg/dL and converted to μmol/L by multiplying by 59.5) were accessible for 288,649 European participants, with no overlap with the UK Biobank dataset. Since multiple published GWASs provide estimates for the same variant‐outcome associations, these estimates can be aggregated through meta‐analysis. Replication analyses were conducted using summary genetic association data from the GUGC Consortium [38].

#### 2.2.3. GWAS Data for Metabolites

We employed the latest and most comprehensive GWAS datasets available for the human metabolome [39]. Utilizing data from the Canadian Longitudinal Study on Aging (CLSA) cohort, researchers investigated 1091 blood metabolites and 309 metabolite ratios, analyzing a total of 8299 participants and around 15.4 million SNPs. The complete summary statistics for the 1400 biomarkers were made publicly accessible.

### 2.3. Data Analysis

#### 2.3.1. Genetic Correlation Analysis

Genome‐wide LDSC was employed to evaluate the genetic associations between sex hormones and serum urate levels or gout risk (https://github.com/bulik/ldsc) [24]. Detailed information for LDSC is available in Supporting Methods. *p* values less than 0.05 were interpreted as indicative of a potential genetic correlation. All statistical analyses were conducted using LDSC Version 1.0.1.

#### 2.3.2. Univariable MR

We conducted two‐sample MR analyses using various methods, including inverse variance weighted (IVW), weighted median, and MR‐Egger approaches. For our primary analysis, we employed the random‐effects inverse‐variance weighted (IVW‐RE) method [40]. This method calculates causal estimates for risk factors by aggregating the Wald ratio estimates from individual SNPs; specifically, each estimate is derived from the ratio of the SNP–outcome association to the SNP–exposure association. Univariable MR analyses were carried out for both sex‐combined and sex‐stratified groups. To address multiple testing, we applied the Benjamini–Hochberg false discovery rate (FDR) adjustment.

To strengthen the robustness of the MR estimates, we further performed a meta‐analysis by combining the results of primary and duplicate analysis. Random effects meta‐analysis was performed across MR estimates obtained from primary and replication analyses. Heterogeneity among MR results was assessed using the Cochran’s chi‐square test and quantified with the *I* ^2^ statistic. *I* ^2^ values of 25%, 50%, and 75% indicate low, moderate, and high levels of heterogeneity, respectively. Figure 1 presents an overview of our MR design. Detailed information for SNPs selecting, statistical power, and sample overlap is available in Supporting Methods.

#### 2.3.3. Sensitivity Analyses

For sensitivity analyses, we utilized several methods, including weighted median, MR‐Egger [41], MR‐PRESSO [42], Radial MR analysis [43], and Causal Analysis Using Summary Effect Estimates (CAUSE), to further assess the robustness of the results. Detailed information for every sensitivity analyses methods is available in Supporting Methods. Additionally, a Steiger directionality test was conducted to eliminate potential reverse causality [44]. Finally, leave‐one‐out analyses were performed to identify any influential SNPs that might bias the associations.

#### 2.3.4. Multivariable MR

For each of our sex hormone exposures, we conducted a Mendelian Randomization Phenome‐Wide Association Study (MR‐PheWAS) focusing on the top 10 SNPs associated with hormones, identified after clumping using the “ieugwasr” R‐package with a significance threshold of 1 × 10^−5^. For the significant findings, we subsequently performed a multivariable Mendelian randomization (MVMR) analysis for each sex hormone, adjusting for relevant pathways by incorporating their summary statistics into the multivariable framework. The selection criteria for genetic instruments in each GWAS dataset remained consistent with those outlined previously.

#### 2.3.5. Bayesian Colocalization Analysis

We employed this method to determine whether two associated traits shared common causal variants based on the included IVs. Detailed information for colocalization analysis is available in Supporting Methods. A PPH4 exceeding 75% was interpreted as suggestive evidence for a causal genetic variant influencing both traits.

#### 2.3.6. Possible Results and Explanations

As illustrated in Figure 1, we categorized three potential outcomes and tiers by integrating findings from genetic correlation, MR, and colocalization analyses based on their effects and levels of statistical significance. The results from genetic correlation and MR analyses were both statistically significant and direct, whereas colocalization analysis yielded only statistically significant results. The MR results comprehensively incorporated the primary analysis using the IVW‐RE method, effectively addressing potential pleiotropic bias.

The specific results and interpretations were as follows: (*Tier 1*) when all three analyses were significant and aligned in direction, this indicated strong genetic evidence for a causal association; (*Tier 2*) when both genetic correlation and MR analyses were significant and aligned, this suggested a robust causal association without shared causal genetic variants; and (*Tier 3*) when MR and colocalization analyses were significant, this was interpreted as weak genetic evidence for a causal association.

### 2.4. Mediation MR

To explore the underlying mechanism between sex hormones and serum urate/gout with strong causal genetic evidence, we performed mediation analyses to estimate the proportion of the effect of sex hormones on serum urate/gout that was attributable to variation in levels of blood metabolites. Detailed information is available in Supporting Methods.

### 2.5. Statistical Analyses

All statistical analyses were conducted using PLINK Version 1.9 and the packages “TwoSampleMR (Version 0.5.7),” “MRPRESSO (Version 1.0),” “Mendelian‐Randomization (Version 0.7.0),” “ieugwasr (Version 1.0.2),” “MVMR (Version 0.4),” “RadialMR (Version 1.1),” “CAUSE (Version 1.2.0.335),” “meta (Version 6.5–0),” “coloc (Version 5.2.3),” and “forestplot (Version 3.1.1)” of the free available statistical software R (Version 4.3.1; R Foundation for Statistical Computing).

## 3. Results

### 3.1. Genetic Correlation of Sex Hormones With Serum Urate and Gout

We first evaluated the shared heritability of sex hormones with serum urate and gout using cross‐trait LDSC among total populations (Figure 2(a)). Generally, sex hormones showed moderate genetic correlation with serum and gout. Specifically, ToT and SHBG showed significant inverse genetic correlation with serum urate (ToT: *r*_*g*_ = −0.143, [95% CI: −0.210, −0.075], and *p* = 3.31 × 10^−5^; SHBG: *r*_*g*_ = −0.155, [95% CI: −0.219, −0.092], and *p* = 1.76 × 10^−6^) and gout (ToT: *r*_*g*_ = −0.154, [95% CI: −0.244, −0.065], and *p* = 7.54 × 10^−4^; SHBG: *r*_*g*_ = −0.137, [95% CI: −0.204, −0.071], and *p* = 4.80 × 10^−5^). Also, BioT showed significant positive genetic correlation with serum urate (*r*_*g*_ = 0.136, [95% CI: 0.080, 0.192], and *p* = 1.80 × 10^−6^) and gout (*r*_*g*_ = 0.136, [95% CI: 0.068, 0.203], and *p* = 8.57 × 10^−5^).

FIGURE 2Genetic correlations of sex hormones with serum urate and gout using linkage disequilibrium score regression (LDSC). (a) Genetic correlations of sex hormones with serum urate and gout in total population. (b) Genetic correlations of sex hormones with serum urate and gout in males. (c) Genetic correlations of sex hormones with serum urate and gout in females. Colors represent the magnitude of genetic correlation of each sex hormones (total testosterone, bioavailable testosterone, SHBG, estradiol, age at menarche, and age at menopause) with serum urate and gout using LDSC; red for positive genetic correlation and blue for negative genetic correlation. Numbers represent the genetic correlation. ^∗^ *p* < 0.05; ^∗∗^ *p* < 0.01; and ^∗∗∗^ *p* < 0.001. Abbreviations: SHBG, sex hormone‐binding globulin.

**Figure:**
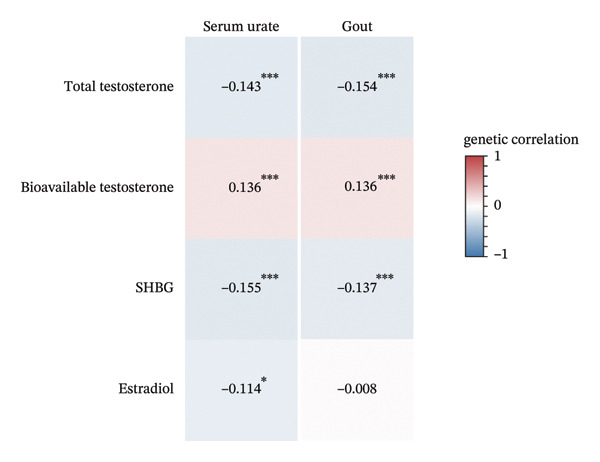
(a)

**Figure:**
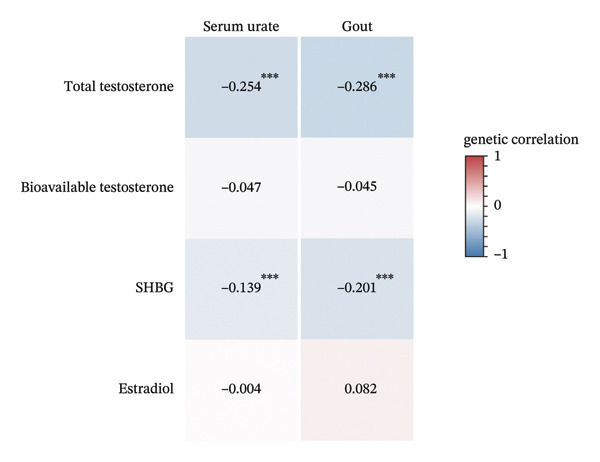
(b)

**Figure:**
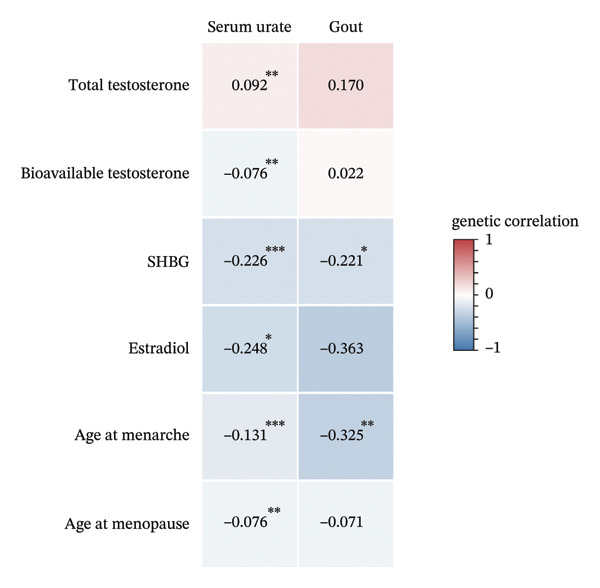
(c)

We next conducted sex‐stratified genetic correlation to further explore if the observed significant genetic correlation was attributed to sexual dimorphism (Figures 2(b) and 2(c)). Generally, results from sex‐stratified genetic correlation analysis were consistent with the overall genetic correlation showing significant genetic correlation of ToT and SHBG with serum urate (ToT: *r*_*g*_ = −0.254, [95% CI: −0.324, −0.185], and *p* = 5.63 × 10^−13^; SHBG: *r*_*g*_ = −0.139, [95% CI: −0.207, −0.072], and *p* = 5.38 × 10^−5^) or gout (ToT: *r*_*g*_ = −0.286, [95% CI: −0.383, −0.189], and *p* = 7.96 × 10^−9^; SHBG: *r*_*g*_ = −0.201, [95% CI: −0.293, −0.109], and *p* = 1.77 × 10^−5^) among men. Among women, all sex hormones showed significant genetic correlation with serum urate (ToT: *r*_*g*_ = 0.092, [95% CI: 0.034, 0.150], and *p* = 0.002; BioT: *r*_*g*_ = −0.076, [95% CI: −0.129, −0.022], and *p* = 0.006; SHBG: *r*_*g*_ = −0.226, [95% CI: −0.294, −0.157], and *p* = 8.76 × 10^−11^; age at menarche: *r*_*g*_ = −0.131, [95% CI: −0.173, −0.089], and *p* = 8.15 × 10^−10^; and age at menopause: *r*_*g*_ = −0.076, [95% CI: −0.128, −0.023], and *p* = 0.005), expect that estradiol showed marginally significant correlation with serum urate (*r*_*g*_ = −0.248, [95% CI: −0.442, −0.054], and *p* = 0.012). Additionally, significant negative genetic correlation of age at menarche with gout among women was observed (*r*_*g*_ = −0.325, [95% CI: −0.523, −0.128], and *p* = 0.001).

### 3.2. The Causal Association Between Sex Hormones and Serum Urate/Gout According to MR Analyses

Then, we used two‐sample MR analysis to develop evidence for causality in the relationship of sex hormones with serum urate and gout. The numbers of IVs ranged from 2 to 205, the F‐statistics for all traits under consideration varied between 912 and 331,995, and the statistical power varied from 4% to 100% (Additional File 1: Table S3 [27]). All F‐statistics exceeded 10, indicating no potential weak instrument bias.

To increase the statistical power of MR analysis, we combined two large‐scale GWAS summary data of serum urate and gout form European ancestry, respectively. According to our meta‐analyses of IVW data, sex hormones had no significant associations with serum urate in European population (all *p* > 0.05; Figure 2(a), Additional File 1: Table S4 [27]). The sensitivity analysis also supported this results (Additional File 1: Table S10 [27]). We noted that genetic liability to ToT was associated with lower risk of gout (OR, 0.602; 95% CI, 0.420–0.861; *p* = 0.005; and FDR *p* = 0.022) (Figure 2(b), Additional File 1: Table S5 [27]). And the association remained significant in weighted median method. Steiger directionality test results indicated that all causal directions were correct. Meanwhile, heterogeneity was observed for ToT, BioT, and SHBG with serum urate and gout (Additional File 1: Tables S4 and S5 [27]). According to MR‐Egger intercept and MR‐PRESSO method, evidence of horizontal pleiotropy was identified for ToT and gout (Additional File 1: Table S5 [27]). After removing outliers, there was still significant association between ToT and risk of gout using IVW method (OR, 0.690; 95% CI, 0.535–0.890; and *p* = 0.004), and the association remained significant in weighted median method (Additional File 1: Table S11 [27]).

As depicted in Figures 3 and 4 and Additional File 1: Table S6–S9 [27], we noted that genetic liability to ToT was associated with increased level of serum urate in the female population (*β*, 0.035; 95% CI, 0.006–0.063; *p* = 0.018; and FDR *p* = 0.018) (Figure 4(a), Additional File 1: Table S7 [27]). However, no such associations were observed in males (Figure 3(a), Additional File 1: Table S6 [27]). In addition, in females, age at menarche and age at menopause showed an inverse causal association with serum urate (age at menarche: *β*, −0.047; 95% CI, −0.074 to −0.020; *p* = 0.001; and FDR *p* = 0.002; age at menopause: *β*, −0.006; 95% CI, −0.012 to −0.001; *p* = 0.032; and FDR *p* = 0.047) (Figure 4(a), Additional File 1: Table S7 [27]). Steiger directionality test results indicated that all causal directions were correct. Significant heterogeneity or pleiotropy was observed in the above results. After removing outliers, there was still significant association between ToT, age at menarche, and age at menopasue in females but not in males (Additional File 1: Tables S12–S15 [27]).

FIGURE 3Forest plot of the results of IVW analysis and colocalization analysis for the association of sex hormones with serum urate (a) and gout (b) in total population. The average value of PPH4 across all regions was taken as the final colocalization result. Abbreviations: ToT, total testosterone; BioT, bioavailable testosterone; SHBG, sex hormone‐binding globulin; FDR, false discovery rate; SNPs, single‐nucleotide polymorphisms; PP, posterior probability.

**Figure:**
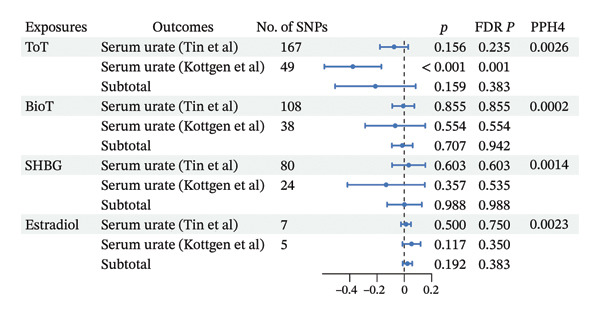
(a)

**Figure:**
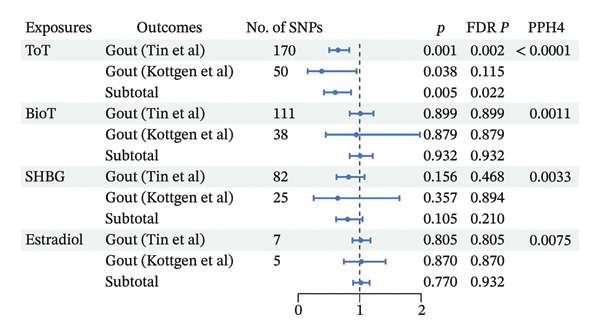
(b)

FIGURE 4Forest plot of the results of IVW analysis and colocalization analysis for the association of sex hormones with serum urate (a) and gout (b) among men. The average value of PPH4 across all regions was taken as the final colocalization result. Abbreviations: ToT, total testosterone; BioT, bioavailable testosterone; SHBG, sex hormone‐binding globulin; FDR, false discovery rate; SNPs, single‐nucleotide polymorphisms; PP, posterior probability.

**Figure:**
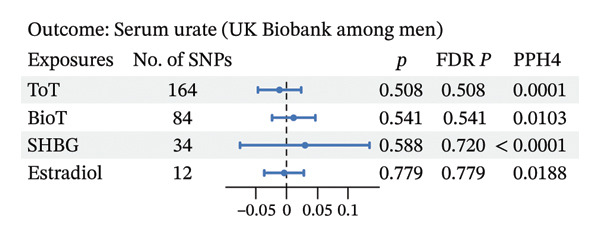
(a)

**Figure:**
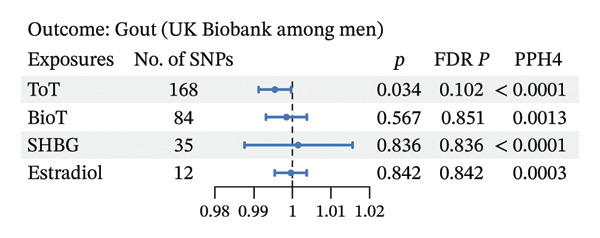
(b)

Furthermore, the leave‐one‐out analysis demonstrated the robustness of the results (Additional File 2: Tables S1–S18 [27]), and sensitivity analysis by MR‐CAUSE showed that ToT remained in a causal association with increased serum urate for females; the causal model of ToT on serum urate was superior to the shared ToT model on serum urate for females (Additional File 1: Table S23).

### 3.3. MVMR Analyses

We conducted a MR‐PHEWAS focusing on the top 10 SNPs independently associated with each sex hormone (Additional File 1: Table S16 [27]). These genetic variants exhibited strong correlations with various concentrations of other sex hormones. Additional phenotypic traits related to the leading genetic variants in the MR‐PHEWAS included BMI, white blood cell count, and sex hormones (ToT and SHBG).

After adjusting for BMI, white blood cell count, and SHBG/ToT, the MVMR analysis suggested there is no evidence for an effect of ToT on gout in total populations (Additional File 1: Table S17 [27]). However, adjusting for these confounders, there was evidence for an inverse effect of ToT on serum urate in females (*β*, −0.110; 95% CI, −0.191 to −0.028; and *p* = 0.008) (Additional File 1: Table S18 [27]). And MVMR analysis demonstrated that there was no evidence for an effect of age at menarche and age at menopause on serum urate in females (Additional File 1: Tables S19–S20 [27]).

### 3.4. Colocalization Analyses

Colocalization analysis revealed no shared causal variant supporting the associations between sex hormones and serum urate or gout in European populations, with the exception of serum estradiol’s association with gout in females (Figures 3, 4, and 5). Furthermore, the PPH4 values for each region are detailed in Additional File 1: Tables S21–S22 [27]. These colocalization results indicate that there may not be a common biological mechanism linking sex hormones to serum urate or gout, aside from estradiol.

FIGURE 5Forest plot of the results of IVW analysis and colocalization analysis for the association of sex hormones with serum urate (a) and gout (b) among women. The average value of PPH4 across all regions was taken as the final colocalization result. Abbreviations: ToT, total testosterone; BioT, bioavailable testosterone; SHBG, sex hormone‐binding globulin; FDR, false discovery rate; SNPs, single‐nucleotide polymorphisms; PP, posterior probability.

**Figure:**
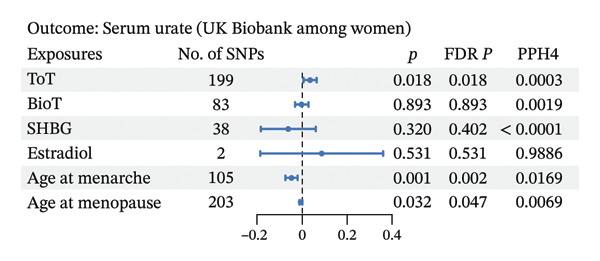
(a)

**Figure:**
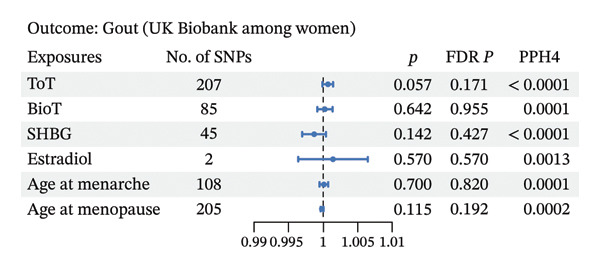
(b)

### 3.5. Mediation Analyses

Only four metabolites, [androstenediol (3β,17β) disulfate (2), 5alpha‐androstan‐3beta 17alpha‐diol disulfate, glycochenodeoxycholate glucuronide (1), 5alpha‐androstan‐3beta, and 17beta‐diol monosulfate (2)], were found to be associated with both ToT and serum urate in females. Furthermore, we found only androstenediol (3β,17β) disulfate (2) as a mediator in the mediation effect analysis. We observed that ToT had an indirect effect on the total effect of serum urate in females (*β*, 0.009; 95% CI, 0.002–0.017; and *p* = 0.009) through androstenediol (3β,17β) disulfate (2) level, with a mediation proportion of 28.4% (Figure 6).

FIGURE 6Graphical representation of Mendelian randomization estimate for (a) “Total effect” of total testosterone on serum urate levels among women and (b) “mediated effect” of total testosterone by androstenediol (3beta, 17beta) disulfate (2) levels on serum urate levels among women. Abbreviations: ToT, total testosterone.

**Figure:**
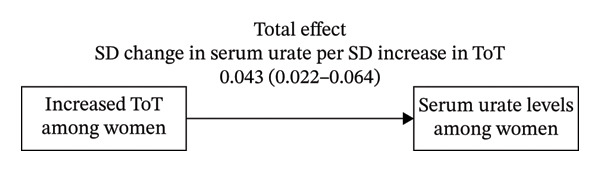
(a)

**Figure:**
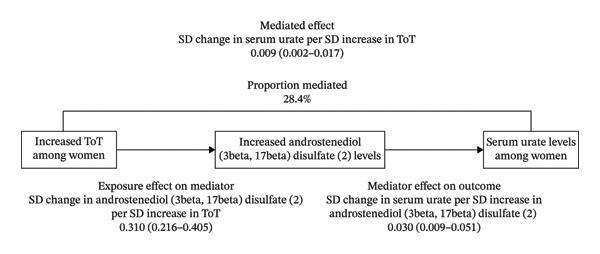
(b)

## 4. Discussion

This is the first study that utilizes genetic correlation, sex‐stratified MR, and colocalization to analyze the association between sex hormones and serum urate/gout. After fully balancing the genetic evidence, we used the latest and largest datasets to investigate the causal associations of genetically determined sex hormones with serum urate and gout across European populations. Among women, we found evidence for associations between serum ToT and increased levels of serum urate. And this result was supported by the results of LDSC and colocalization analyses. Moreover, our findings suggest that ToT may increase serum urate by modulating metabolites, with androstenediol (3β,17β) disulfate (2) potentially playing a mediating role—accounting for approximately 28.4% of the association between ToT and serum urate among women.

Our findings were partially inconsistent with previous MR study results. Previous MR studies have focused on examining associations of genetically predicted sex hormones with risk of gout [45] and various health outcomes [46]. Specifically, Jiang et al. found that one‐unit higher log‐transformed TT was associated with a 52% (95% CI, 0.39–0.58) lower risk of gout in males, which is opposite to free testosterone (OR, 1.74 and 95% CI, 1.38–2.20) and BioT (OR, 1.78 and 95% CI, 1.41–2.25) [45]. Yuan et al. also proved that higher TT appeared to lower the risk of gout in males. These results are consistent with our analysis among men but have no statistical significance after FDR adjusted. The discrepancies observed between these studies may stem from the utilization of varying summary‐level GWAS data for exposures and outcomes. In contrast, we leveraged the most recent and largest GWAS summary data available from European populations. Furthermore, we integrated genetic evidence from multiple methodologies.

Although the causal association between genetically predicted total testosterone and serum urate was observed exclusively among women, the underlying biological mechanisms remain incompletely understood. From a physiological perspective, serum urate homeostasis is primarily determined by a balance between urate production and extrarenal clearance, particularly renal tubular handling and intestinal excretion [47]. Accumulating evidence suggests that sex hormones modulate urate levels predominantly through the regulation of urate transport and reabsorption rather than through direct effects on purine metabolism [48].

Experimental and epidemiological studies have indicated that estrogens exert a urate‐lowering effect, partly by enhancing urate excretion via regulation of urate transporters. For example, estradiol has been shown to upregulate intestinal ABCG2 expression through the PI3K/Akt signaling pathway, thereby facilitating extrarenal urate elimination [7]. However, evidence from renal models indicates that estrogen replacement in ovariectomized mice may suppress ABCG2 expression in the kidney [49], suggesting tissue‐specific and context‐dependent effects. These seemingly contradictory findings may be explained by the presence of estrogen response elements in the upstream promoter region of the ABCG2 gene, which may differentially regulate transporter expression across tissues [50].

In contrast, the role of androgens in urate metabolism appears more heterogeneous and sex dependent. Dehydroepiandrosterone (DHEA), an abundant adrenal steroid hormone, has been shown to activate mineralocorticoid receptors and inhibit glucocorticoid receptors, thereby reducing renal urate excretion and increasing serum urate levels [51]. Animal studies further suggest that testosterone can upregulate the mRNA and protein expression of sodium‐dependent monocarboxylate transporter 1 (SMCT1 and SLC5A8), which cooperates with urate transporter 1 (URAT1) to facilitate proximal tubular urate reabsorption and contribute to HUA [49]. However, observational evidence in humans has been inconsistent. For example, a prospective cohort study of adult men reported that lower testosterone levels were associated with an increased risk of HUA [16]. These discrepancies highlight the potential influence of detection bias, residual confounding, and pleiotropy in conventional observational studies, underscoring the value of genetic approaches.

The female‐specific association observed in our study may reflect fundamental differences in androgen physiology between sexes. In women, circulating testosterone concentrations are substantially lower, and androgenic activity is largely determined by peripheral metabolism and conversion of steroid precursors rather than direct gonadal secretion [52]. Consequently, downstream androgen metabolites may play a more prominent biological role than total testosterone itself. This hypothesis is supported by our mediation MR analysis, which identified androstenediol (3β,17β) disulfate (2) as a partial mediator of the association between ToT and serum urate, accounting for approximately 28.4% of the total effect.

Androstenediol (3β, 17β) disulfate is a sulfated androgen metabolite derived from testosterone and DHEA, representing a stable circulating reservoir of biologically active androgens [53]. Sulfated steroids can undergo tissue‐specific desulfation, thereby exerting local hormonal effects that may not be fully captured by circulating total testosterone levels [54]. It is, therefore, plausible that androstenediol sulfate more accurately reflects the biologically relevant androgenic milieu influencing renal or intestinal urate transport in women. Although direct experimental evidence remains limited, this metabolite may modulate urate homeostasis through androgen receptor–mediated regulation of urate transporters, alterations in tubular reabsorption, or indirect effects on inflammatory and oxidative pathways involved in urate production and clearance.

Importantly, the partial mediation observed in our analysis suggests that testosterone influences serum urate through multiple, parallel pathways. In addition to steroid metabolites, these may include direct hormonal effects on urate transporters, interactions with estrogen signaling, and broader metabolic processes related to insulin resistance and inflammation [55]. The absence of a comparable mediation effect in men further supports the hypothesis that sex‐specific endocrine environments shape distinct biological pathways linking sex hormones to urate metabolism. More studies are needed to clarify the potential mechanisms through which sex hormones may exert effects on serum urate and gout.

Our study has several notable strengths. The genetic correlation, MR, and colocalization analyses each possess unique strengths and limitations that can partially complement one another, helping to reduce the risk of false negatives and false positives. This work is a significant contribution to the field as it offers guidance on integrating evidence from genetics‐driven studies accumulated to date, facilitating a more reliable interpretation of the epidemiological relationship between sex hormones and serum urate or gout. Additionally, we utilized the latest available GWAS data, which included the largest sample sizes for both exposure and outcome investigations, and we verified our findings through various methodologies and across different ancestries. As a result, our objective estimates of the associations between sex hormones and serum urate/gout may provide greater precision than previous estimates and are less susceptible to bias.

However, we acknowledge the limitations of our study. First, a fundamental assumption of MR is that SNPs are not associated with any confounders affecting the exposure or the outcome. Although we accounted for pleiotropy bias, it is impossible to completely eliminate the risk of pleiotropic bias in any MR study. Second, we examined three exposed phenotypes and utilized different sets of IVs, which resulted in varying statistical power across our analyses, potentially introducing weak IV bias. Moreover, while colocalization analysis of genes and proteins is a widely used technique, it remains in the developmental stage for establishing optimal thresholds. We included all IVs in our colocalization analysis and set a cutoff value of 0.75 for PPH4. Additionally, although we drew from multiple data sources to enhance statistical power, the relatively small number of patients limited the power of our MR analyses. Furthermore, despite utilizing data from large genetic studies, our study lacked the capacity to detect very small effects. We also could not adjust for potential confounders due to the absence of individual‐level data. Finally, given the limited GWAS data available for the European population, our results may not be applicable to Asian populations. Thus, we advise caution when interpreting our findings in racially and ethnically diverse contexts.

## 5. Conclusion

In summary, our comprehensive analyses of genetic correlation, MR, and colocalization indicate that higher concentrations of ToT are linked to elevated serum urate levels in women of European ancestry. This finding suggests that hyperandrogenism may contribute to the development of HUA in this population.

## Author Contributions

**Shiwei Li**: data curation (equal); formal analysis (equal); investigation (equal); methodology (equal); software (equal); visualization (equal); and writing–original draft (lead). **Mengjuan Zhang**: data curation (equal); formal analysis (equal); validation (equal); and writing–original draft (equal). **Xuemei Wang**: data curation (equal); formal analysis (equal); methodology (equal); and writing–original draft (equal). **Yadi Huang**: investigation (equal) and validation (equal). **Bo Huang**: software (equal) and visualization (equal). **Meng Wang**: methodology (equal) and software (equal).**Ming Liu**: writing–original draft (supporting); writing–review and editing (equal); supervision (equal); and project administration (supporting). **Jingqiu Cui**: conceptualization (lead); supervision (equal); writing–review and editing (equal); project administration (lead); and funding acquisition (lead).

## Funding

This work was supported by the National Natural Science Foundation of China (grant no. 82070854), the Tianjin Key Medical Discipline (Specialty) Construction Project (TJYXZDXK‐3‐002C), and the Tianjin Medical University Clinical Special Disease Research Center ‐ Neuroendocrine Tumor Clinical Special Disease Research Center.

## Ethics Statement

All GWASs included in this study were approved by the respective institutional review boards and ethical committees, and all participants provided signed informed consent.

## Conflicts of Interest

The authors declare no conflicts of interest.

## Supporting Information

Additional supporting information can be found online in the Supporting Information section.

The following Supporting information are provided along with this manuscript:

## Supporting information


**Supporting Information 1** Supporting Table 1: Table S1: STROBE Statement–Checklist of items relevant to Mendelian randomization studies. Table S2: Characteristics of the summary GWAS datasets used in this study. Table S3: Summary of instrumental variables (IVs) selected for Mendelian randomization analyses. Tables S4–S9: Mendelian randomization estimates for the causal associations between sex hormones and serum urate or gout risk in the total population, and stratified by sex (men and women). Tables S10–S15: Sensitivity analyses using various MR methods (e.g., MR‐Egger, weighted median, and MR‐PRESSO) for each exposure‐outcome pair in total population and sex‐stratified groups. Table S16: MR‐PHEWAS results for the top 10 SNPs of each exposure using a significance threshold of *p* < 1 × 10^−5^. Tables S17–S20: Multivariable MR (MVMR) estimates for the association between total testosterone, age at menarche/menopause, and gout or serum urate levels in total or sex‐stratified populations. Tables S21–S22: Bayesian colocalization analysis for the shared genetic basis between sex hormones and serum urate or gout. Table S23: Results from MR‐CAUSE analyses exploring the liability effect of total testosterone on serum urate levels in females.

**Supporting Information 2** Supporting Table 2: Leave‐one‐out analysis.

**Supporting Information 3** Supporting Methods: A detailed description of the statistical methods used in the study, including procedures for LDSC regression, Mendelian randomization (univariable and mediation MR), sensitivity analyses (e.g., MR‐Egger, MR‐PRESSO, Radial MR, and MR‐CAUSE), and Bayesian colocalization.

## Data Availability

The data that support the findings of this study are openly available in figshare at https://doi.org/10.6084/m9.figshare.27629487. Summary statistics for GWAS are publicly available for download. All statistical code in the article is available upon request.
