# Supplementary material for: Genetic Association Between Sex Hormones, Serum Urate, and Gout: A Comprehensive Mendelian Randomization Study
Source: Int J Endocrinol. 2026 Apr 20;2026:8839727. doi: 10.1155/ije/8839727 (PMC13092932; doi:10.1155/ije/8839727)
Supplement: Supplementary file 3 — Supporting Information 3 Supporting Methods: A detailed description of the statistical methods used in the study, including procedures for LDSC regression, Mendelian randomization (univariable and mediation MR), sensitivity analyses (e.g., MR‐Egger, MR‐PRESSO, Radial MR, and MR‐CAUSE), and Bayesian colocalization. [file IJE-2026-8839727-s003.docx]

**Genetic association between sex hormones, serum urate and gout: a comprehensive Mendelian randomization study**

Shiwei Li ^1*^, Mengjuan Zhang ^1*^, Xuemei Wang ^1*^, Yadi Huang ^1^, Bo Huang ^1^, Meng Wang ^1^, Ming Liu ^1 #^, Jingqiu Cui ^1 #^

^1^ Department of Endocrinology and Metabolism, Tianjin Medical University General Hospital, Tianjin 300052, China.

^*^ These authors were co-senior authors and contributed equally to this work.

**Supplementary Methods**

1. **Linkage disequilibrium score regression (LDSC)**

The LDSC method calculates genetic correlation by taking into account the influence of all SNPs, including those that do not reach genome-wide significance. We excluded SNPs that were not aligned with HapMap3 SNPs and those with a minor allele frequency below 0.01. The results are reported as genetic correlation (rg) along with standard error (SE). It is important to note that LDSC analysis results may not be available if one or both traits display low heritability [1]. *P* values less than 0.05 were interpreted as indicative of a potential genetic correlation. All statistical analyses were conducted using LDSC version 1.0.1.

1. **Univariable MR**

For each of the sex hormone GWASs, we limited the genetic variants to those with minor allele frequencies of at least 1%, ensuring they were bi-allelic and located on autosomal chromosomes. We selected SNPs associated with the exposure at the genome-wide significance level (*P*<5×10^−8^). Independence of SNPs was assessed using stringent criteria (*r^2^*, 0.001; clumping window, 10000 kb). If an instrumental SNP for the exposure was not available in the outcome data set, we replaced it with a suitable proxy SNP (*r^2^*>0.8 in the European 1000 Genomes Project reference panel using LDlink [https://ldlink.nci.nih.gov/]) or removed it in the absence of such a proxy. We harmonized the SNP alleles across studies and removed palindromic SNPs with ambiguous allele frequencies (0.42–0.58). *R^2^* was estimated from effect allele frequency (EAF) and the SNPs-exposure association (*β*) as the sum of $2\times EAF\times(1-EAF)\times\beta^{2}$. And we calculated SNP-specific F-statistics by the formula $F=\frac{(N-2)\times R^{2}}{1-R^{2}}$. The F-statistics for all traits under consideration exceeded 10, indicating no potential weak instrument bias [2].

In addition, statistical power was estimated from the sample size, proportion of cases, and *R^2^* using the web tool “mRnd” (https://shiny.cnsge nomics.com/mRnd/)[3]. The risk of bias from sample overlap was estimated by using an online tool (<https://sb452.shinyapps.io/overlap/>) [4].

1. **Sensitivity analyses**

The weighted median approach generates causal estimates from the median of the weighted empirical density function of each SNP's effect estimates, provided that at least 50% of the instrumental variants are valid [5]. In the MR-Egger method, the slope coefficient represents the valid causal estimate in the presence of pleiotropy [6]. If the intercept of the MR-Egger regression deviates from zero with a *P*-value less than 0.05, it indicates the presence of pleiotropic SNPs. Due to various methodological limitations, including violations of the InSIDE assumption and the effects of outlier variants, the MR-Egger method was not considered efficient for estimating causal effects in our analysis [7]. The MR-PRESSO method was applied to conduct a global test for pleiotropy, with outliers assessed using the MR-PRESSO outlier test. This framework estimates the causal effect using IVW methods after the removal of outliers [8]. We also implemented Radial MR to identify the presence of outliers, setting a threshold *P*-value of 0.05; any detected outliers were removed, and Radial MR was reapplied until no outliers remained. After excluding these SNPs, sensitivity analyses were repeated [9]. Furthermore, to mitigate potential pleiotropy, we employed MR-CAUSE analysis to identify causal relationships while accounting for both correlated and uncorrelated horizontal pleiotropic effects [10]. In CAUSE analyses, gamma represents the effect size of exposure on the outcome, eta indicates the effect size of correlated pleiotropy, Q denotes the proportion of variants showing correlated pleiotropy, and P reflects the probability of accepting a sharing model. We used Cochran’s Q statistic (derived from IVW method) and I-squared metrics (*I^2^*) to assess the heterogeneity among the multiple instrumental variants, with *P*-value less than 0.05 in Cochran’s Q and *I^2^* values greater than 50% indicating significant heterogeneity [11].

1. **Bayesian colocalization analysis**

Five mutually exclusive hypotheses were evaluated: (1) there is no causal genetic variant for either trait (H0); (2) there is one causal genetic variant for trait 1 only (H1); (3) there is one causal genetic variant for trait 2 only (H2); (4) there are two distinct causal variants, one for each trait (H3); and (5) there is a shared causal variant for both traits (H4). The posterior probability (PP) quantifies the support for each hypothesis and is represented as PPH0, PPH1, PPH2, PPH3, and PPH4 [12]. We defined regions with 500 kb windows both upstream and downstream of each instrumental variable for the MR analysis, and the average PPH4 value across all regions was taken as the final colocalization result. A PPH4 exceeding 75% was interpreted as suggestive evidence for a causal genetic variant influencing both traits.

**5. Mediation MR**

In mediation analyses, the effect of sex hormones on serum urate/gout was considered the “total effect”. The “mediated effect” was defined as the effect of sex hormones on serum urate/gout that was mediated through levels of the corresponding blood metabolites [13]. This mediated effect was estimated using the product method, whereby the beta estimate for the association of sex hormones with the respective blood metabolites was multiplied by the beta estimate for the association of the blood metabolites with serum urate/gout [13]. Standard errors were estimated using the propagation of errors method. The “proportion mediated” was calculated by dividing the indirect effect by the total effect, and the 95% CI was estimated using the propagation of errors method. The proportion mediated reflects the percentage of the total effect of sex hormones on serum urate/gout that is mediated by the respective blood metabolites.

**References**

1. Bulik-Sullivan BK, Loh PR, Finucane HK, Ripke S, Yang J, Patterson N, et al. LD Score regression distinguishes confounding from polygenicity in genome-wide association studies. Nat Genet. 2015;47(3):291-5. Epub 20150202. doi: 10.1038/ng.3211. PubMed PMID: 25642630; PubMed Central PMCID: PMCPMC4495769.

2. Burgess S, Thompson SG, Collaboration CCG. Avoiding bias from weak instruments in Mendelian randomization studies. Int J Epidemiol. 2011;40(3):755-64. Epub 2011/03/19. doi: 10.1093/ije/dyr036. PubMed PMID: 21414999.

3. Burgess S. Sample size and power calculations in Mendelian randomization with a single instrumental variable and a binary outcome. International Journal of Epidemiology. 2014;43(3):922-9. doi: 10.1093/ije/dyu005.

4. Burgess S, Davies NM, Thompson SG. Bias due to participant overlap in two‐sample Mendelian randomization. Genetic Epidemiology. 2016;40(7):597-608. doi: 10.1002/gepi.21998.

5. Bowden J, Davey Smith G, Haycock PC, et al. Consistent Estimation in Mendelian Randomization with Some Invalid Instruments Using a Weighted Median Estimator. Genetic Epidemiology. 2016;40(4):304-14. doi: 10.1002/gepi.21965.

6. Bowden J, Davey Smith G, Burgess S. Mendelian randomization with invalid instruments: effect estimation and bias detection through Egger regression. Int J Epidemiol. 2015;44(2):512-25. Epub 2015/06/08. doi: 10.1093/ije/dyv080. PubMed PMID: 26050253; PubMed Central PMCID: PMCPMC4469799.

7. Burgess S, Thompson SG. Interpreting findings from Mendelian randomization using the MR-Egger method. Eur J Epidemiol. 2017;32(5):377-89. Epub 2017/05/21. doi: 10.1007/s10654-017-0255-x. PubMed PMID: 28527048; PubMed Central PMCID: PMCPMC5506233.

8. Verbanck M, Chen CY, Neale B, et al. Detection of widespread horizontal pleiotropy in causal relationships inferred from Mendelian randomization between complex traits and diseases. Nat Genet. 2018;50(5):693-8. Epub 2018/04/25. doi: 10.1038/s41588-018-0099-7. PubMed PMID: 29686387; PubMed Central PMCID: PMCPMC6083837.

9. Lin F, Shi Y, Song W, Weng Y, Zou X, Chen X, et al. Daytime napping and the incidence of Parkinson's disease: a prospective cohort study with Mendelian randomization. BMC Med. 2024;22(1):326. Epub 20240813. doi: 10.1186/s12916-024-03497-7. PubMed PMID: 39135019; PubMed Central PMCID: PMCPMC11321229.

10. Morrison J, Knoblauch N, Marcus JH, Stephens M, He X. Mendelian randomization accounting for correlated and uncorrelated pleiotropic effects using genome-wide summary statistics. Nat Genet. 2020;52(7):740-7. Epub 20200525. doi: 10.1038/s41588-020-0631-4. PubMed PMID: 32451458; PubMed Central PMCID: PMCPMC7343608.

11. Bowden J, Del Greco MF, Minelli C, Davey Smith G, Sheehan N, Thompson J. A framework for the investigation of pleiotropy in two-sample summary data Mendelian randomization. Stat Med. 2017;36(11):1783-802. Epub 20170123. doi: 10.1002/sim.7221. PubMed PMID: 28114746; PubMed Central PMCID: PMCPMC5434863.

12. Giambartolomei C, Vukcevic D, Schadt EE, Franke L, Hingorani AD, Wallace C, Plagnol V. Bayesian test for colocalisation between pairs of genetic association studies using summary statistics. PLoS Genet. 2014;10(5):e1004383. Epub 20140515. doi: 10.1371/journal.pgen.1004383. PubMed PMID: 24830394; PubMed Central PMCID: PMCPMC4022491.

13. Carter AR, Sanderson E, Hammerton G, Richmond RC, Davey Smith G, Heron J, et al. Mendelian randomisation for mediation analysis: current methods and challenges for implementation. Eur J Epidemiol. 2021;36(5):465-78. Epub 2021/05/08. doi: 10.1007/s10654-021-00757-1. PubMed PMID: 33961203; PubMed Central PMCID: PMCPMC8159796.
